# The Diagnostic Efficiency of 99mTc-EDDA/HYNIC-Octreotate SPECT-CT in Comparison with 111In-Pentetrotide in the Detection of Neuroendocrine Tumours

**DOI:** 10.4274/Mirt.68552

**Published:** 2013-12-10

**Authors:** Emel Koçyiğit Deveci, Meltem Ocak, Murat Fani Bozkurt, Selcan Türker, Levent Kabasakal, Ömer Uğur

**Affiliations:** 1 Department of Nuclear Medicine, Hacettepe University, Ankara, Turkey; 2 Department of Pharmaceutical Technology, Faculty of Pharmacy, Istanbul University, Istanbul, Turkey; 3 Department of Radiopharmacy, Faculty of Pharmacy, Hacettepe University, Ankara, Turkey; 4 Department of Nuclear Medicine, Cerrahpasa Faculty of Medicine, Istanbul University, Istanbul, Turkey

**Keywords:** Somatostatin receptors, neuroendocrine tumors, Single–Photon Emission Computerized Tomography, Computeized Tomography, X ray

## Abstract

**Objective:** The aim of this study was to assess the diagnostic efficiency of ^99m^Tc-EDDA/HYNIC-Octreotate in comparison with ^111^Inpentetrotide scintigraphy in the detection of neuroendocrine tumors. This study also evaluates the impact of SPECT-CT hybrid imaging on somatostatin receptor scintigraphy (SRS) interpretation and clinical management of these tumors.

**Methods:** Fourteen patients were included in the study. All patients underwent a whole body and SPECT-CT imaging with both ^99m^Tc- EDDA/HYNIC-octreotate and ^111^In-pentetrotide. Images were evaluated both visually and semiquantitatively.

**Results:** On patient basis, the diagnostic results of both studies were similar. The number of lesions detected by ^99m^Tc- EDDA/HYNICOctreotate were higher than the number of lesions detected by ^111^In-pentetrotide however the difference was not significant (40/43( 93%), 36/43 (83%) p=0.109). Semiquantitative analysis showed higher tumor/organ count ratios for both whole-body and SPECT ^99m^Tc- EDDA/HYNIC-Octreotate scans.

**Conclusion:** The results of this study suggested that, ^99m^Tc- EDDA/HYNIC-Octreotate may be a better alternative to ^111^In- pentetrotide due to high image quality and lower radiation dose. SPECT/CT is a valuable tool for the assessment of neuroendocrine tumors by providing the precise anatomic localization of scintigraphic findings thus improving lesion detectability and characterization.

**Conflict of interest:**None declared.

## INTRODUCTION

Neuroendocrine tumors (NET) are a wide group of tumors which can involve different organs and systems. In the diagnosis, treatment and follow up of these tumors different common features such as expression of unique receptors, possession of special metabolic pathways, synthesis and secretion of different materials in peptide and amine structure are utilized. Imaging of NET can be done by various methods of nuclear medicine. The most commonly used target is somatostatin receptors. 

The first somatostatin analog designed as a radiopharmaceutical was ^111^In-DTPA-octreotide (^111^In-pentetreotide, Octreoscan®) (1). However, high radiation exposure, production cost of In-^111^, and the difficulties in supplying are its limitations. Beside these, waiting period up to 48 hours for imaging after the injection hampers patient compliance. 

Somatostatin analogs labeled with Technetium-99m(^99m^Tc) provide a new perspective in somatostatin receptor imaging. First results showed that ^99m^Tc labeled somatostatin analogs has a sensitivity and specificity equivalent to ^111^In-pentetreotide. Moreover, in some studies it was found to be even better than ^111^In-pentetreotide in terms of success in lesion detection. Among ^99m^Tc labeled somatostatin analogs ^99m^Tc-depreotide (P-829) was the first radiopharmaceutical which was proven to be valuable for the evaluation of solitary pulmonary nodules (2,3). The other radiopharmaceutical was ^99m^Tc-HYNIC-Tyr3 octreotide (^99m^Tc-HYNIC TOC) which was granted marketing authorization and made available for the wide use. ^99m^Tc-HYNIC-Tyr3-octreotate (^99m^Tc-HYNIC-TATE) is another analogue which differs from ^99m^Tc-HYNIC TOC with terminal amino acid in the peptide sequence and showed higher receptor affinity and better internalization (4). Moreover, it was reported that ^99m^Tc-HYNIC-TATE has higher affinity to somatostatin receptor subtype-2 than octreotide (5). 

Tc-99m has lower energy than In-^111^ which enables higher dose administration, better image quality and suitable radiation dosimetry. Higher doses of ^99m^Tc also increase the image quality and lesion detection capability of SPECT imaging. Availability as an instant cold kit and labeling with ^99m^Tc obtained from an onsite generator enables imaging of the patients on the same day of admission to the Nuclear Medicine departments. Radioguided surgery of neuroendocrine tumors with ^99m^Tc labeled somatostatin analogs is also more successful compared to radioguided surgery with In-^111^ because of higher injected dose of the tracer and better physical properties of ^99m^Tc for the detection with a gamma probe. 

Ga-68 labeled PET tracers -in particular ^68^Ga-DOTATOC, ^68^Ga-DOTATATE, and ^68^Ga-DOTANOC- have been shown to have higher detection rate compared with conventional SRS and diagnostic CT (6). However, both ^68^Ge/^68^Ga generators and PET scanners are still lower in number than ^99^Mo/^99^Tc generators and SPECT cameras. 

In this study we evaluated the accuracy of Tc-99m labelled EDDA/HYNIC-Octreotate using SPECT-CT and compared the results with ^111^In-pentetreotide scintigraphy in the same patient population.

## MATERIALS AND METHODS

**Patients**

This study has been approved by the Local Ethic Committee and was supported in part by Turkish Scientific and Research Council (TUBITAK) project no: 109S498 and 109S101. Fourteen patients (7 men, and 7 women) with diagnosis of NET who were referred to Nuclear Medicine Department for somatostatin receptor scintigraphy (SRS) were included in the study. All patients gave their informed consent and underwent SRS with ^99m^Tc-EDDA/HYNIC-Octreotate and ^111^In-pentetreotide. 

**Radiopharmaceuticals**

^111^In-pentetreotide was obtained as a commercial kit (Covidien, Netherland). Radiochemical purity analyzed by TLC exceeded 95% using the recommended instant TLC method with 0.1 mol/L citrate buffer, pH 5, as solvent. 

^99m^Tc-EDDA/HYNIC-octreotate was reconstituted from a kit produced at Istanbul University Radiopharmacy laboratory. It was prepared from a freeze-dried kit formulation (formulated by using CHRIST ALPHA 2-4 LSC freeze drier) which was described by von Guggenberg et.al. (7) Briefly, ^99m^Tc-HYNIC-TATE was prepared from kit containing 1:20 µg HYNIC-TATE, 20 µg SnCl2.2H2O, 10 mg EDDA (ethylenediaminediacetic acid), 20 mg of tricine and 50 mg of mannitol and in Vial 2: 35.6 mg Na2HPO4.2H2O/mL (0.2 M solution) in vial 1. The contents of Vial 1 were dissolved in 1 mL of Vial 2 and 1 mL of fresh ^99m^Tc-eluate containing up to 1.5 GBq was immediately added. The mixture was heated in a boiling water bath for 10 minutes. In order to purify both the ^99m^Tc labeled peptides, Sep- Pak C18 cartridges (Waters, USA) were used. The reaction mixture was applied to a Sep-Pak C 18 Column which had been pre-washed with 99% ethanol and saline. The column was then gradually washed with saline (2.5 mL) and 50 % ethanol (0.5 mL) through sterile membrane filter into the sterile vial under aseptic conditions. Administration of radiopeptide to the patient was done after SPE purification. Instant thin-layer chromatography on silica gel (ITLC-SG, Gelman Sciences) was performed using different mobile phases. 0.1 M sodium citrate pH 5 to determine the nonpeptide-bound ^99m^Tc-coligand and ^99m^TcO4 -(Rf=1), methanol/1 M ammonium acetate 1/1 for ^99m^Tc-colloid (Rf=0). Rf values for the radiolabeled peptide were 0.0, and 0.8, 1.0, respectively. ITLC determination and quantification has been done by using a Cyclon Phosphor Imager (Perkin Elmer). Radiochemical purity results exceeded 95%. 

**Imaging with ^111^In-pentetreotide**

After intravenous administration of 222-250 MBq of ^111^In-pentetreotide, whole body, planar and SPECT/CT imaging at 4th and 24th hours were carried out using double-headed hybrid gamma camera system integrated with x-ray CT (Infinia VC and Hawkeye, GE Medical Systems, U.S.A). Scintigraphic images were acquired with medium energy, parallel hole collimator, set to 172 and 245 keV energy peak, with 20% energy window and CT imaging was acquired in 4.5-5 minutes by taking simultaneous multiple slices in 512x512 matrix in helical mode on, by using 2,5 mA current at 140 keV, provided that the beam scope is 2 cm in each gantry rotation. After CT acquisition, SPECT images were recorded in a protocol of 128x128 matrix, 15-20 seconds/frame rotation with an angle of 360 degree. 

Imaging with ^99m^Tc-EDDA/HYNIC Octreotate was performed within 5-15 days after ^111^In pentetreotide imaging. After intravenous administration of 370-400 MBq of ^99m^Tc- EDDA/ HYNIC octreotate, whole body, planar and SPECT-CT imaging at fourth hour were carried out using double headed hybrid gamma camera system integrated with X-Ray CT (Infinia VC and Hawkeye, GE Medical Systems, U.S.A). Scintigraphic images were acquired with low energy high resolution parallel hole collimator peaked at 140 keV with a 10% energy window. CT images were acquired in 4.5-5 minutes by taking simultaneous multiple slices in 512x512 matrix in helical mode on, by using 2,5 mA current at 140 keV, provided that the beam scope is 2 cm in each gantry rotation. After CT acquisition, SPECT images were recorded in a protocol of 128x128 matrix, 15-20 seconds/frame rotation with an angle of 360 degree.

The image reconstruction was performed using iterative reconstruction (OSEM iteration numbers 2 and 10) on a Xeleris® work station (GE Medical Systems). 

**Scintigraphic Evaluation**

The planar and SPECT-CT images of ^99m^Tc-EDDA/HYNIC Octreotate and ^111^In pentetreotide studies were separately evaluated and compared to each other. 

In the visual evaluation, the areas that showed higher uptake than the background were accepted as pathological uptake. CT component of hybrid imaging was used correlatively to verify the localization and characterization of the pathological uptake in scintigraphic studies. 

Regions of interests (ROIs) were drawn over the areas which showed pathological uptake in scintigraphic studies for semiquantitative analysis. Identical ROIs were also placed over liver, spleen and right thigh in order to calculate background counts. For semiquantitative analysis of SPECT images, transaxial slices were used, where identical ROIs were placed over areas which showed pathological uptake as well as over liver and kidney and over the area with the least counts in that particular transaxial slice to represent the background. From these calculations tumor/liver, tumor/kidney, tumor/spleen, tumor/thigh, tumor/background (for transaxial images of SPECT) activity ratios were obtained. The ratios which were obtained from planar and SPECT images in both imaging studies were compared to each other. 

The contribution of planar, SPECT and SPECT-CT images to the diagnosis were separately assessed. The uptake patterns were scored according to whether it can be exactly localized or not. Also another scoring scale was used to assess the intensity of uptake as (0: no suspicious uptake in the imaging area, 1: it is enough to differ between suspicious uptake/physiologic uptake, 2: it is not enough to differ between suspicious uptake/physiologic uptake). 

**Statistical Analysis**

For the measurable variables (tumor uptake counts, scintigraphic uptake ratios), the correlation between the values was evaluated by using correlation test between independent variables (Spearman test), the assessment of the difference between values were evaluated by the significance of difference between dependent variables (Wilcoxon test).

The assessment of the difference between different imaging methods was performed by using the significance of the difference between dependent variables test (McNemar test).

For all statistical tests that were used, p<0.05 value was accepted as statistically significant. 

## RESULTS

Indication for SRS imaging was for the staging of five patients who had histopathological NET diagnosis and restaging residual disease/relapse evaluation of 9 patients who had been previously diagnosed and treated. The primary tumor was located in lungs in two patients, in gastrointestinal system in 10 patients, in neck in one patient and in kidney in the rest one patient (Table 1).

All patients were examined by one or more of the other radiological imaging methods which were given in Table 2.

The histopathological diagnosis were bronchial carcinoid, well differentiated neuroendocrine neoplasm, paraganglioma, poorly-differentiated neuroendocrine carcinoma and carcinoid tumor. The serum biochemical analyses were given in Table 2.

**Comparison of the Radiopharmaceuticals in Terms of Tumor Uptake**

**Figure 2A-B:**
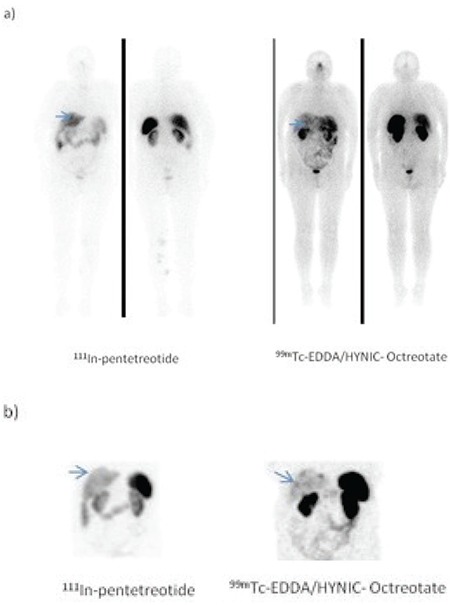
70-year-old woman with liver metastases of well-differentiatedneuroendocrine tumor after resection and radiofrequency ablationof the primary lesion. a) planar b) SPECT images of 111In pentetreotideand 99mTc EDDA/HYNIC-Octreotate SRS. Liver metastases were detectedby 99mTc EDDA/HYNIC-Octreotate imaging which are indicated by arrowsbut not seen clearly with 111In-pentetreotide planar and SPECT images.

The patient-based visual comparison of the tumor uptake of radiopharmaceuticals revealed that all patients who showed pathological uptake with ^111^In-pentetreotide imaging also showed pathologic uptake in the same region with ^99m^Tc-EDDA/HYNIC-Octreotate imaging (Figure1 A,B). One patient (patient no: 9) who had no pathologic uptake with, ^111^In-pentetreotide displayed pathological uptake on ^99m^Tc-EDDA/HYNIC-Octreotate planar and SPECT images (Figure 2 a,b). ^99m^Tc-EDDA/HYNIC-Octreotate imaging results were highly concordant with ^111^In-pentetreotide imaging. When the concordance between the images with two radiopharmaceuticals were examined in terms of capability of detecting pathological uptake, Spearman correlation factor was found to be 62% (good), which was statistically significant (p<0.05).

The lesion-based comparative analysis revealed that scintigrapy detected 40 out of 43 lesions which were detected with anatomical imaging (contrast enhanced diagnostic CT and MRI together).

Thirty six of 40 lesions (83%) were shown with both ^99m^Tc-EDDA/HYNIC-Octreotate, and ^111^In-pentetreotide. However, 4 lesions were only visualised with ^99m^Tc-EDDA/HYNIC-Octreotate. Although the number of tumoral uptake sites were more for ^99m^Tc-EDDA/HYNIC-Octreotate compared to ^111^In-pentetreotide the difference was not statistically significant (p=0.109). Three lesions were only visualized with anatomical imaging (contrast enhanced diagnostic CT and MRI together). Two of these lesions were cervical lymph nodes which were reported as suspected lymph nodes containing calcifications. Another lesion was in the liver but MRI could not distinguish whether the lesion was a metastatic focus or fibrotic area. SRS results were negative for these lesions. But these foci were not confirmed by histopathological examination.

The tumor uptake ratios on both ^99m^Tc-EDDA/HYNIC-Octreotate and ^111^In pentetreotide studies were compared with each other using semiquantitative analysis. In planar images, the tumor/liver and tumor/kidney uptake ratios were significantly higher with ^99m^Tc-EDDA/HYNIC-Octreotate compared to ^111^In-pentetreotide (p=0.015 and p=0.003, respectively Wilcoxon test). On the other hand, the difference between the tumor/spleen and tumor/thigh uptake ratios with ^99m^Tc-EDDA/HYNIC-Octreotate and ^111^In-pentetreotide were not significant (p=0.121 and p=0.245 respectively, Wilcoxon test).

In SPECT images, the tumor/liver, tumor/kidney, tumor/spleen, tumor/ background uptake ratios with ^99m^Tc-EDDA/HYNIC-Octreotate were significantly higher than ^111^In pentetreotide ratios (p=0.015, p=0.007, p=0.025 and p=0.066, respectively, Wilcoxon test).

**The contribution of ^99m^Tc-EDDA/HYNIC-Octreotate to Patient Management**

**Figure 3:**
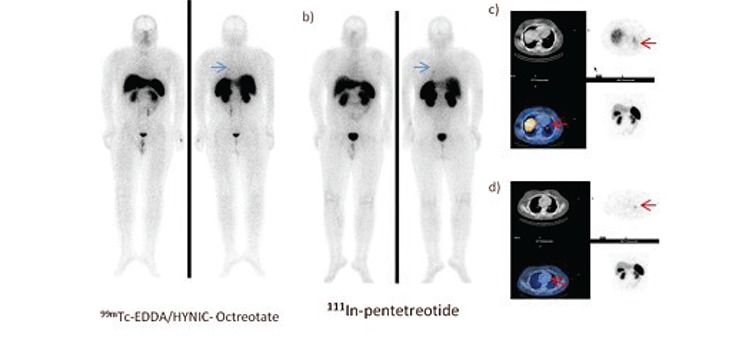
18-year-old man with the diagnosis of ectopic Cushing’s syndrome a) 99mTc- EDDA/HYNIC-Octreotate, b) 111In-pentetreotide images of SRS. Metastaticlymph node seen at the left hilar area but primary focus was not detected in the planar images. c) SPECT and SPECT-CT fusion images of the 99mTc-EDDA/HYNIC-Octreotate revealed a foci of pathological tracer uptake in the lower lobe of the left lung (arrows) d) The left hilar lymph node involvement wasseen on SPECT and SPECT-CT fusion images of the 99mTc- EDDA/HYNIC-Octreotate

The contribution of ^99m^Tc-EDDA/HYNIC-Octreotate to diagnosis and treatment were as follows: On a patient with the diagnosis of ectopic Cushing’s syndrome, (Patient no: 1) the left hilar lymph node involvement was seen on whole-body planar images with ^111^In pentetreotide and ^99m^Tc-EDDA/HYNIC-Octreotate. Conventional imaging methods also detected hilar lymph node. On SPECT-CT study, the primary tumor focus was detected on the lower lobe of the left lung with both agents. But conventional imaging methods did not detect the primary focus, most probably due to the localization and anatomical appearance of the lesion. Because the lesion was 10 mm long and not enhanced with contrast which was located at the basal lobe of the lung. After 185 MBq ^99m^Tc-EDDA/HYNIC Octreotate administration, the patient underwent gamma probe-guided surgery. The primary tumor focus and the hilar lymph node were successfully removed. As a result of orientation to the lesion promptly, surgery and anesthesia time was relatively reduced. Compared to pre-planned area, a significant contribution was achieved to patient morbidity and mortality by excising much smaller area (Figure 3).

**Figure 4:**
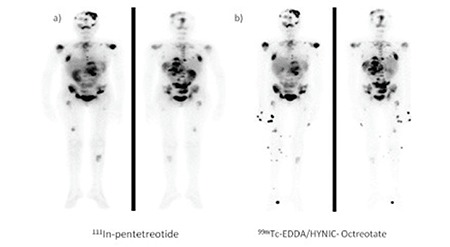
19-year-old female with dissemination of paraganglioma after resection of the primary lesion. a) 111In-Octreoscan and b) 99mTc-EDDA/HYNIC-OctreotateSRS . Wide spread metastatic lesions were detected by both agents. The foci at the right lower extremity were thought to be due to contamination

Following the ^99m^Tc-EDDA/HYNIC-Octreotate imaging studies, ^177^Lu-Octreotate treatment was suggested for four patients who were diagnosed with extensive tumor involvement of the whole body, and ^177^Lu-Octreotate treatment was applied for two patients who (gave consent) accepted (Figure 4). 

## DISCUSSION

Neuroendocrine tumors are heterogenous family of neoplasms that present with a wide range of morphologic, functional and behavioural characteristics (8). Because NETs have ability to express specific receptors on their cell membrane, such as somatostatin receptors, functional imaging plays an important role for both diagnosis and follow up of these tumors (9). Researchers are working to develop new somatostatin analogues labeled with different radionuclides to get higher diagnostic yield. ^99m^Tc-labeled somatostatin analogs give a new perspective on somatostatin receptor imaging. In our study, a patient based comparison revealed that ^99m^Tc-EDDA/HYNIC-Octreotate has a similar biodistribution with that of ^111^In-pentetreotide, which is consistent with the results of other studies (10). Patients who had tumoral uptake with ^111^In-pentetreotide imaging had also ^99m^Tc-EDDA/HYNIC-Octreotate uptake within the same location.

A lesion based comparison of two agents revealed that ^99m^Tc-EDDA/HYNIC-Octreotate showed higher number of tumoral involvement especially in the liver (Patient 9).

Similarly other studies in the literature also reported that more metastatic lesions are detected in the liver using ^99m^Tc labeled somatostatin analogs as compared to ^111^In-pentetreotide (10,11). However, the results of our study are not statistically significant probably due to small patient group.

Having ideal imaging characteristics, ^99m^Tc presents an advantage for SRS imaging compared to ^111^In which gives better results with hybrid imaging system SPECT-CT for correct characterization and correct localization of pathological lesions. The correct anatomic location will eliminate false positive results, and improves the diagnostic accuracy. On our study, SPECT-CT detection efficiency of the anatomical localization was significantly higher than SPECT study.

**Figure 5:**
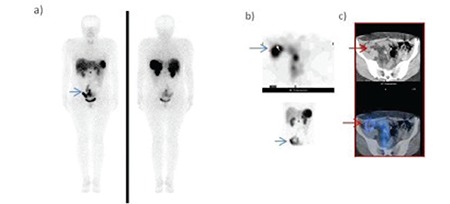
34-year-old woman with metastases after resection of primary focus which was in the ileum. a) Whole body planar images revealed pathologicaltracer uptake in the lower part of the abdomen (arrows). b) The same area was seen with SPECT images but distinguishing physiologicaluptake from tumoral involvement was not possible. c) SPECT-CT fusion images revealed that the involvement was due to physiological bowel activity

SPECT-CT studies yielded more accurate information in particular the involvement of the abdominal region (12) (Figure 5).

Semiquantiative analysis for the evaluation of tumoral involvement revealed that tumor/organ uptake ratios with ^99m^Tc-EDDA/HYNIC-Octreotate on both planar and SPECT studies were higher than ^111^In-pentetreotide. It was reported that Octreotate has a higher receptor affinity and internalization capacity due to its terminal threoninol (13). In vitro studies that were done with SS receptor-positive cell culture showed that octreotate chelated with DTPA or DOTA shows 14-17 times more affinity to SStr2 than octreotide, and 8-10 times greater affinity than TOC (5). Due to these features, TATE is also preferred for treatment studies (14). The higher tumor/non-tumor uptake ratios also provide an advantage for imaging one the same day (15). In limited studies, ^99m^Tc-EDDA/HYNIC-Octreotate was shown to be a promising agent for both imaging and gamma-probe applications (16,17).

In this study we showed that ^99m^Tc-EDDA/HYNIC-Octreotate is a good alternative for SRS with the advantages of better pharmacokinetic properties, lower radiation dose and higher diagnostic accuracy especially when hybrid imaging methods are used.

## Figures and Tables

**Table 1:**
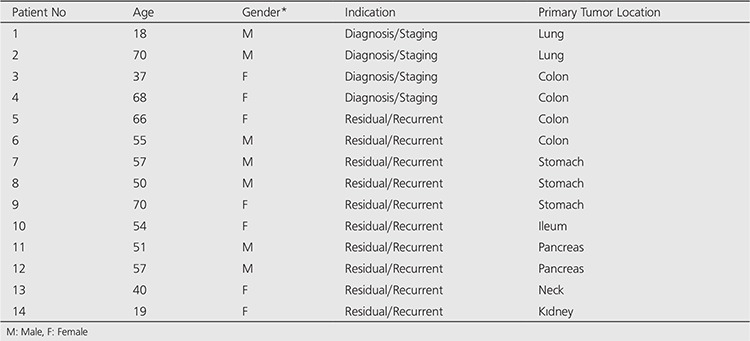
The Demographic characteristics and primary tumor localizations of the patients

**Table 2:**
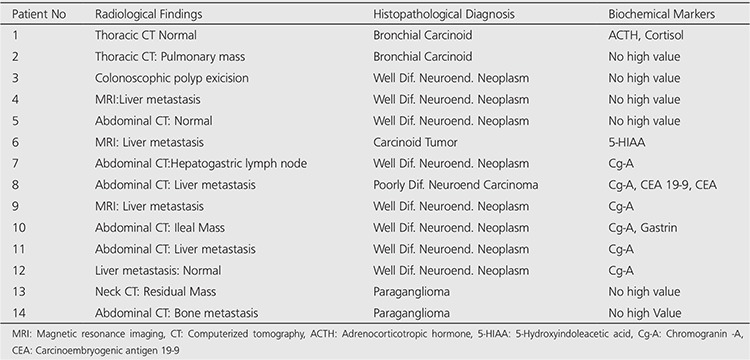
The Radiological Findings, histopathological diagnosis and biochemical markers of the patients

**Figure 1-A:**
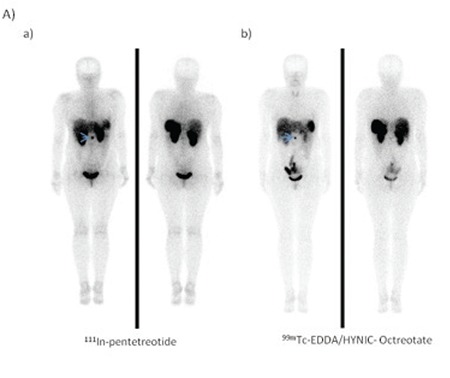
34-year-old woman with a metastatic focus after resection ofprimary tumor which was in the ileum. a) 111In-Pentetreotide and b) 99mTcEDDA/HYNIC-Octreotate SRS revealed the same lesion (arrows).

**Figure 1-B:**
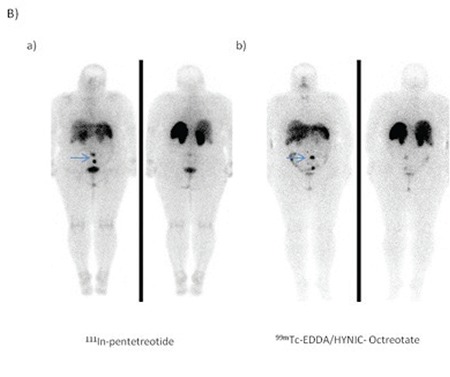
54-year-old woman after resection of a primary tumor ofwell-differentiated neuroendocrine carcinoma. a) 111In- Pentetreotide andb) 99mTc EDDA/HYNIC-octreotate SRS revealed two foci of pathologicaltracer uptake in the lower part of the abdomen (arrows).
